# Applying a Combination of the YOLOv8 Model and 3D Point Cloud Images in Asphalt Pavement Maintenance

**DOI:** 10.3390/s26102938

**Published:** 2026-05-07

**Authors:** Yangyang Wang, Shoujing Yan, Weibo Shi, Chenchen Xi, Jiachen Shi, Fengxia Chi, Jintao Wei

**Affiliations:** 1 Department of Architecture and Civil Engineering, Zhejiang University, Hangzhou 310030, China; profxnzhang@126.com; 2 Zhejiang Scientific Research Institute of Transport, Hangzhou 310023, China; swb_wk01@163.com (W.S.); 18356032654@163.com (C.X.); s_jia_chen@163.com (J.S.); chifx2025@163.com (F.C.); weijintao2020@163.com (J.W.)

**Keywords:** 3D laser point cloud image, YOLOv8 model, deep learning networks, image recognition, asphalt pavement maintenance

## Abstract

**Highlights:**

**What are the main findings?**

- YOLOv8-Seg&ASF model demonstrates superior performance, achieving a recall rate exceeding 85% and an average precision of 79.5%, thereby facilitating efficient and accurate identification of multiple distress types, including cracks and potholes.
- Precise extraction of distress features was achieved, with Root Mean Square Errors (RMSE) of 0.974 and 1.687 for crack length and angle measurements, respectively.

**What are the implications of the main findings?**

- The proposed method overcomes the limitations of traditional optical image detection in directly extracting distress dimensional features, thereby providing precise data support for road maintenance.
- It significantly improves the automation and efficiency of pavement distress detection, presenting a novel technical framework for intelligent decision-making in asphalt pavement maintenance.

**Abstract:**

Asphalt pavement distress detection plays a pivotal role in highway maintenance, providing an essential basis for optimizing maintenance strategies and allocating funding. Consequently, quick detection and efficient identification of distress are crucial for enhancing the quality of highway maintenance. This study aims to acquire high-precision distress data using 3D laser point cloud technology, identify distress types via the YOLO algorithm, and extract geometric features such as length and angle. Specifically, a recognition method based on 3D laser point cloud images is proposed, where point cloud data are converted into planar images for processing. Experimental results indicate that the laser point cloud detection achieves millimeter-level precision, the distress recall rate exceeds 85%, and the identification precision reaches 79.5%, demonstrating satisfactory detection accuracy and efficiency.

## 1. Introduction

As a fundamental component of modern transportation infrastructure, asphalt pavement plays a critical role in ensuring traffic safety and maximizing highway operational benefits. Regarding pavement distress detection, optical camera-based image acquisition technologies have gained extensive application, predominantly depending on manual discrimination or neural network models for identification [1,2,3]. Nevertheless, optical images are intrinsically two-dimensional projections, failing to directly retrieve depth and elevation data regarding pavement distresses. This inherent constraint results in considerable discrepancies in the geometric features of distresses derived from pixel-based inference [4,5]. Moreover, traditional detection techniques are characterized by a heavy reliance on manual intervention, which entails low efficiency, significant subjective bias, and elevated costs. Consequently, these methods are ill-equipped to satisfy the escalating requirements for precision and rapidity in highway maintenance.

Three-dimensional laser scanning technology presents a new paradigm for overcoming the limitations mentioned above. This technology acquires high-precision point cloud data via high-speed laser scanning, achieving millimeter-level measurement accuracy, and has exhibited remarkable superiority in highway surveying and bridge modeling [6]. In contrast to optical images, 3D point cloud data facilitate not only the precise identification of fracture distresses (e.g., cracks and potholes) but also the effective capture of the 3D geometric characteristics of deformation distresses (e.g., rutting and subsidence), offering comprehensive data support for maintenance strategies [7,8]. Nevertheless, the deployment of 3D laser point cloud technology in pavement distress detection remains constrained by severe challenges. Specifically, the processing of massive point cloud datasets involves intricate workflows with limited automation, while the standards for distress feature extraction and evaluation are yet to be fully established, hindering the technology’s broader engineering application.

In recent years, substantial advancements have been realized in 3D laser point cloud data processing [9,10], particularly within the realm of 3D point cloud imaging. Addressing the challenge of point cloud segmentation, Roland Billen [11] proposed a voxel-based feature engineering method that effectively characterizes point cluster features, providing robust support for supervised and unsupervised classification. Utilizing a decision tree algorithm for classification, this method achieved an F1-score exceeding 85%. Similarly, Alfan Rizaldy Pratama et al. [12] introduced the Curvature-based Variance Ratio Density-Based Spatial Clustering of Applications with Noise (CVR-DBSCAN) algorithm to analyze object morphology in 3D point cloud images. Experimental simulations involving three stacking conditions—well-separated, well-stacked, and randomly stacked—demonstrated the algorithm’s exceptional stability, attaining a separation accuracy of 98.83%. However, the aforementioned methods predominantly focus on the global segmentation of 3D targets. When applied to pavement distress scenarios—characterized by micro-scale features, unstructured nature, and complex backgrounds—these methods often lack targeted intelligent identification mechanisms. Consequently, this leads to insufficient efficiency and robustness in target extraction.

With the advancement of deep learning technology, methods based on Convolutional Neural Networks (CNNs) have gradually emerged as the mainstream approach for 3D point cloud recognition [13,14,15]. P.L. Chithra et al. [16] proposed a tensor-based efficient decoder for 3D LiDAR point clouds, which significantly reduces the dimensionality of high-order point cloud data by integrating statistical subspace outlier detection with logarithmic transformation techniques. This method compresses spatial information from point cloud images of varying sizes into six bytes, achieving an improved Hausdorff peak signal-to-noise ratio for shortest-distance point cloud images. Similarly, Yourui Huang et al. [17] developed a 3D point cloud segmentation and phenotypic measurement method based on an improved PointNet. By incorporating MogaBlock and a self-attention mechanism into the feature propagation layer, they enhanced both local and global feature extraction. Experimental results demonstrated that this method achieved an Overall Accuracy (OA) of 97.70% and a mean Intersection over Union (mIoU) of 96.01% in rapeseed point cloud segmentation tasks. Despite the remarkable accuracy achieved by deep convolutional neural networks, their intricate architectures lead to prolonged inference times. Consequently, the trade-off between high precision and real-time requirements remains a challenge, hindering the deployment of these models in rapid highway inspection contexts.

However, despite the superior precision of deep convolutional neural network (CNN) models, their detection efficiency is often constrained by high computational costs [18], rendering them suboptimal for rapid pavement distress detection. In recent years, single-stage object detection methods, exemplified by the YOLO model [19,20], have streamlined the detection workflow and significantly enhanced processing speed. Therefore, a critical question arises regarding the limitations of existing techniques, which presents a significant obstacle to achieving a balance between accuracy and efficiency in 3D laser point cloud-based pavement distress detection.

This paper presents a novel pavement distress recognition framework by integrating the YOLO algorithm with 3D laser point cloud imagery. The performance evaluation in this study was conducted in a laboratory environment, where the collected road point cloud data was analyzed and processed using a data-center-grade GPU. The research findings aim to provide efficient distress analysis for pavement maintenance decision-making, rather than real-time vehicle-mounted detection. The structure of the paper is organized as follows: Section 2 describes the test site and details the 3D laser point cloud detection workflow, including instrumentation, testing protocols, data processing, and initial results, and further applies the YOLOv8 model to optimize detection precision and efficiency; Section 3 examines the measurement precision of the 3D laser point cloud system and reports the experimental results of asphalt pavement distress detection and recognition; finally, Section 4 provides the concluding remarks.

## 2. Road Distress Detection Based on 3D Laser Point Cloud Method

### 2.1. Tested Road Section

**Figure 1:**
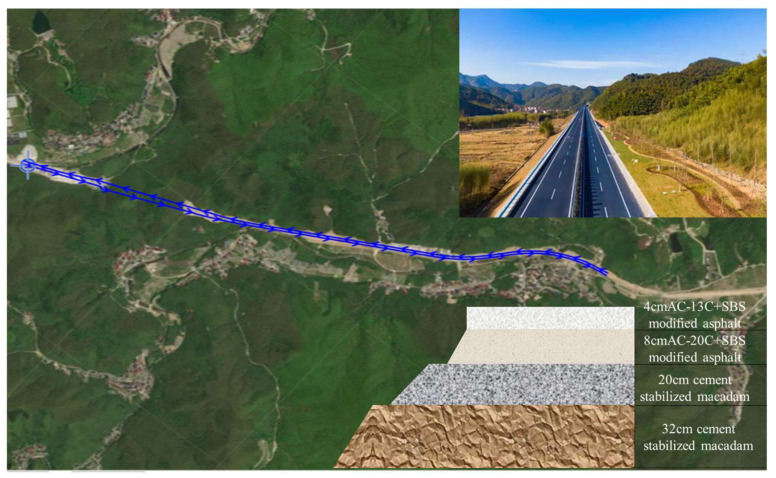
Tested road section.

The tested section selected for this study is located on Provincial Highway S304 in Huzhou, Zhejiang Province. The section extends from stake mark K0+000 to K13+200, covering a total length of 13.2 km. Detailed information regarding the location, pavement structure, and paving materials of the test section is illustrated in Figure 1.

### 2.2. Three-Dimensional Laser Point Cloud Calibration

**Figure 2:**
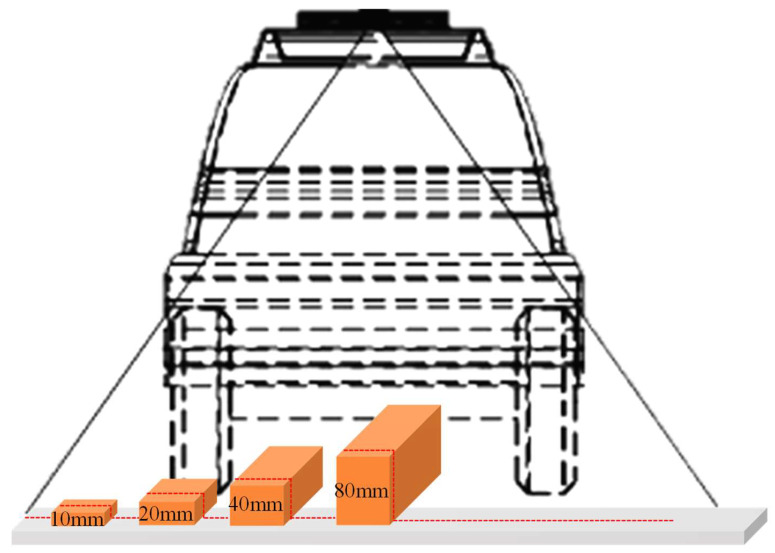
Three-dimensional Laser point cloud height calibration.

**Figure 3:**
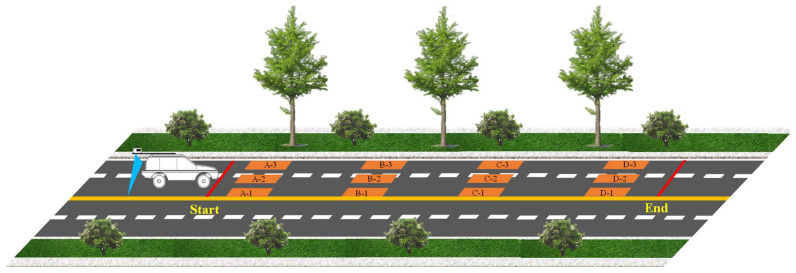
Three-dimensional Laser point cloud planar calibration.

The calibration process for the 3D laser point cloud equipment was conducted with a focus on the following aspects:

(a) The calibration primarily focused on height measurement precision and planar measurement precision, the latter of which encompassed both transverse and longitudinal dimensions. To guarantee a standardized and accurate calibration procedure, the standard gauge blocks utilized in the experiments were traceable to legal metrological verification certificates, with manufacturing tolerances of 0.0004 mm for length, width, and height. For the height and plane measurements, four repeated trials were performed with reloading between each measurement to verify repeatability.
(b) Four types of standard blocks with varying heights—10 mm, 20 mm, 40 mm, and 80 mm—were selected for height measurement calibration; the length and width dimensions of these blocks were 10 mm and 20 mm, respectively. For planar transverse and longitudinal length measurement, four types of standard blocks were employed: Type A (400 mm × 200 mm × 10 mm), Type B (500 mm × 250 mm × 20 mm), Type C (500 mm × 500 mm × 10 mm), and Type D (500 mm × 500 mm × 25 mm).
(c) Standard blocks with heights of 10 mm, 20 mm, 40 mm, and 80 mm were prepared, with one block for each height category. These blocks were sequentially positioned on a flat plate with a spacing of 30 cm, as illustrated in Figure 2. Under static conditions, 3D laser point cloud system measured the heights of the four standard blocks sequentially via time-triggered acquisition to calibrate the height measurement error of the system.
(d) For planar transverse and longitudinal length measurement, three standard blocks of each type (A, B, C, and D) were prepared. The blocks were arranged sequentially in the longitudinal direction with a spacing of 5 m and a transverse spacing of 0.5 m, as illustrated in Figure 3. The 3D laser point cloud system initiated acceleration from the starting point of the test section and successively measured all standard blocks at a speed of 50 km/h. The length and width values for each block were recorded to calibrate the planar measurement error of the system.

### 2.3. Three-Dimensional Laser Point Cloud Planar Imaging

**Figure 4:**
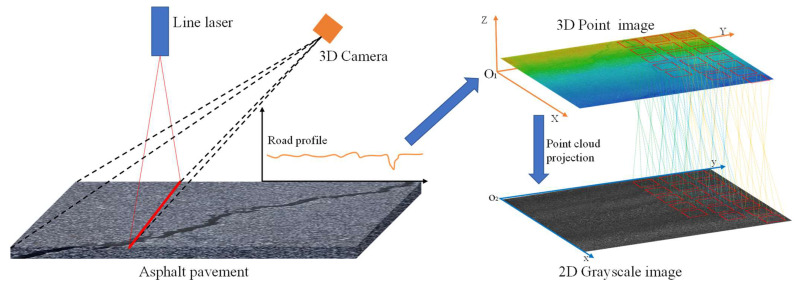
Three-dimensional Laser point cloud planar imaging.

To quantitatively characterize the geometric features of asphalt pavement distress, such as length and width, this study utilized a laser point cloud grayscale compression technique [21,22]. Specifically, three-dimensional laser points were mapped to grayscale values and projected onto a two-dimensional plane to generate a 2D grayscale image, as illustrated in Figure 4.

The conversion from laser point clouds to grayscale images is a two-step process. First, an RGB image is generated from the point cloud according to the elevation variations within a defined region, as formulated in Equation (1).

(1) $$\left\{\begin{array}{l} \tilde{R}=R_{i}h_{i}\frac{h_{\max}-h_{\min}}{h_{\max}}\\ \tilde{G}=G_{i}h_{i}\frac{h_{\max}-h_{\min}}{h_{\max}}\\ \tilde{B}=B_{i}h_{i}\frac{h_{\max}-h_{\min}}{h_{\max}} \end{array}\right.$$

Given a point cloud region with a minimum elevation of $h_{\min}$ and a maximum elevation of $h_{\max}$ the conversion of the point cloud into an RGB image is formulated as shown in Equation (2). Subsequently, the RGB image is transformed into a grayscale image based on the reflectance intensity values of the laser point cloud.

(2) $$Grey=k_{i}\left[\begin{array}{l} \alpha_{1}\\ \alpha_{2}\\ \alpha_{3} \end{array}\right]\times \left[\tilde{R},\tilde{G},\tilde{B}\right]$$

where $\tilde{R},\tilde{G},\tilde{B}$ are the trichromatic channel values of the color image; $h_{\min}$ and $h_{\max}$ are the minimum and maximum elevation values of the point cloud, respectively; $k_{i}$ denotes the point reflectance with a range of 0 to 1; and $\alpha_{1},\alpha_{2},\alpha_{3}$ are trichromatic channel coefficients set to 0.299, 0.587, and 0.114, respectively.

### 2.4. Pavement Distress Recognition Algorithm

#### 2.4.1. Pavement Distress Dataset

Pavement distress can be essentially characterized as anomalous fluctuations in pavement elevation. This phenomenological characteristic is inherently consistent with the fundamental logic of point cloud visualization. Utilizing the method described in Section 2.3, digital images with explicit physical significance were derived, effectively eliminating the uncertainties associated with illumination variations and optical imaging algorithms.

In this study, lane-level high-precision laser scanning systems were employed to acquire laser point cloud samples at high speeds. The acquisition accuracy reached 0.5 mm, covering a total mileage of 50 km with full lane width coverage. Subsequently, a 2D grid array was constructed—where M and N denote the number of grids in the x and y directions, respectively—to facilitate the imaging and dimensionality reduction in the point cloud $P_{M\times N}[x,y,z]$, as formulated in Equations (1) and (2). Ultimately, a depth map (image Z) with a resolution of 4000 × 2560 was generated, corresponding to a physical pixel size of 0.002 m.

**Figure 5:**
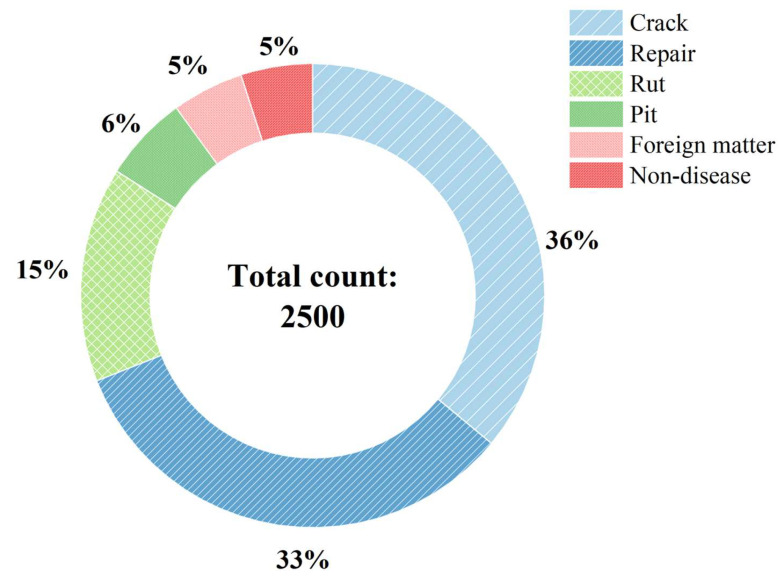
Distribution of target categories in the dataset.

The Z-maps were annotated utilizing the open-source software LabelImg (v1.8.0). The dataset was classified into six distinct categories: cracks, repairs, ruts, potholes, foreign objects, and non-distress regions. A total of 2500 samples were generated in COCO format, with the distribution of each category illustrated in Figure 5.

#### 2.4.2. Model Architecture

In pavement distress detection and segmentation, achieving an optimal trade-off between detection speed and recognition precision is imperative. Consequently, this study adopts the YOLOv8-Seg instance segmentation model [23] as the baseline architecture. This model is well-suited to the simultaneous recognition and segmentation of multi-scale targets, effectively addressing challenges ranging from fine cracks to extensive potholes.

To further enhance cross-granularity object detection performance, this paper employs the Asymptotic Feature Pyramid Network (AFPN) [24] to optimize the YOLOv8-Seg architecture. AFPN adopts a progressive feature fusion strategy, which better addresses the semantic gap between non-adjacent layers (e.g., low-level geometry and high-level semantics). This is particularly crucial for detecting road defects with significant morphological variations, such as fine cracks and large potholes.

The network architecture incorporates an Adaptive Spatial Fusion (ASF) module [25]. Employing a progressive fusion strategy, the module initially integrates low-level features (e.g., P3 and P4), sequentially incorporates higher-level features (e.g., P5), and ultimately synthesizes the top-level features from the backbone. This hierarchical approach effectively mitigates the semantic gap between non-adjacent layers and minimizes information degradation during multi-stage transmission. To address potential feature conflicts at identical spatial locations, an adaptive spatial weight assignment mechanism is utilized within the AFPN. This mechanism dynamically assigns weights to multi-layer features, thereby suppressing contradictory information and preserving salient features.

Specifically, the fused feature vector at position (i,j) of level l is derived through a linear combination of multi-level feature vectors, as formulated in Equation (3).

(3) $$y_{ij}^{l}=\alpha_{ij}^{l}\cdot x_{ij}^{1\to l}+\beta_{ij}^{l}\cdot x_{ij}^{2\to l}+\gamma_{ij}^{l}\cdot x_{ij}^{3\to l}$$

where $x_{ij}^{n\to l}$ denotes the feature vector from level n to level l at position (i,j); $\alpha_{ij}^{l}$, $\beta_{ij}^{l}$ and $\gamma_{ij}^{l}$ represents the spatial weights at position (i,j) of level l for the three levels, respectively. These weights satisfy the normalization constraint $\alpha_{ij}^{l}+\beta_{ij}^{l}+\gamma_{ij}^{l}=1$. The dynamic spatial weights ($\alpha_{ij}^{l}$, $\beta_{ij}^{l}$ and $\gamma_{ij}^{l}$) are generated via the integrated ASF module. Specifically, the concatenated multi-scale features are processed by a lightweight pathway consisting of 1 × 1 convolutions for channel reduction and a subsequent Softmax activation along the channel dimension. This yields dynamic spatial weight maps matching the input resolution. These weights are then applied to their respective feature maps before element-wise addition, effectively achieving spatially adaptive fusion.

**Figure 6:**
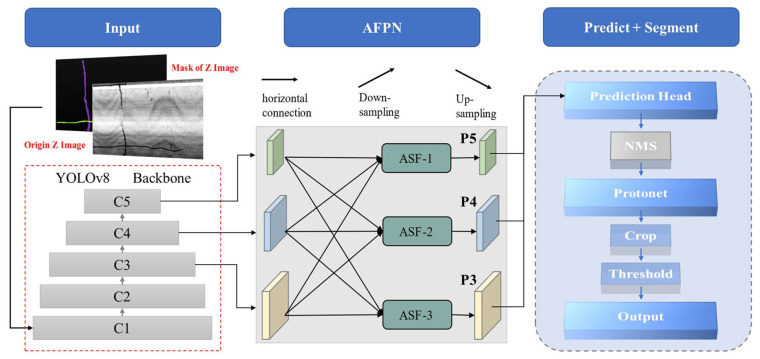
YOLOv8-Seg&ASF model architecture.

In the model construction phase, the backbone of the original YOLOv8 architecture is preserved. Its output is connected to the AFPN module via a 1 × 1 convolution to standardize the channel dimensions of P3, P4, and P5. Bilinear interpolation is employed for upsampling. Conversely, a differentiated downsampling strategy is adopted, utilizing convolution kernels and strides of varying sizes: specifically, 2 × 2, 4 × 4, and 8 × 8 kernels with strides of 2, 4, and 8 are used to achieve 2×, 4×, and 8× downsampling, respectively. Following the feature fusion process (i.e., the ASF operation), four consecutive residual blocks are appended; each block consists of two 3 × 3 convolutional layers, consistent with the ResNet architecture. Finally, the fused tensor undergoes a 3 × 3 convolution (stride = 1, padding = 1) to mitigate aliasing effects before being fed into the detection and segmentation heads. The overall architecture of the proposed model is illustrated in Figure 6.

#### 2.4.3. Model Training

The dataset was partitioned into training, validation, and test sets at a ratio of 70%:15%:15%. The YOLOv8-Seg&ASF model, configured with a size of ‘L’, was initialized using the official Ultralytics YOLOv8-Seg pre-trained weights. The model was trained for 300 epochs with an input resolution of 1000 × 640. Prior to training, three data augmentation techniques—vertical flip, horizontal flip, and Mosaic augmentation—were applied with equal probability. To facilitate convergence, the backbone weights were frozen during the initial 15% of epochs, restricting the optimization to the AFPN module and the detection heads.

Hyperparameters for model training are as follows: a batch size of 16, the AdamW optimizer, an initial learning rate of 0.001, and a final learning rate factor of 0.01. The loss function is consistent with that of the original YOLOv8-seg model. The dataset partition-ing scheme of 70% training, 15% validation, and 15% testing was adopted, and 5-fold cross validation was introduced to ensure the robustness of the results.

The model implementation and training were conducted using the PyTorch (v2.4.1) framework. The experiments were performed on a workstation equipped with an Intel Core i9-12900K CPU, an NVIDIA RTX A6000 GPU (48 GB VRAM), and 128 GB of RAM.

### 2.5. Pavement Distress Feature Extraction Method

After completion of object detection, bounding boxes and classification labels for various pavement distresses were obtained. Subsequently, this study focused on extracting the geometric features—specifically length and angle—of crack and repair targets.

To quantify these parameters, it is necessary to derive the skeletal contour line of the target from the Z-map. This process comprises three sub-tasks: first, extracting the morphological skeleton of the closed region; second, identifying connected components to calculate the length; and third, fitting a minimum bounding ellipse to the distress region, utilizing the ellipse’s rotation angle to determine the orientation. It should be emphasized that the proposed second-order central moment-based ellipse fitting method is strictly constrained to single-linear cracks (i.e., longitudinal and transverse cracks). Complex topologies such as branched or alligator cracks are beyond the scope of this current study and require skeletonization-based vector analysis, which we reserve for future work.

#### 2.5.1. Extracting Length Features

The road distress region can be cropped using the bounding box coordinates generated by the YOLO model [26,27,28]. Since the data is derived from point clouds (i.e., the aforementioned Z-map), each distress region is represented as a single-channel intensity image. While significant elevation disparities exist between road distress and background regions, defining a universally applicable gradient operator for elevation segmentation remains challenging. Furthermore, the bounding boxes output by the YOLO model may encompass multiple distress instances or overlapping targets; therefore, clustering post-processing is required to isolate and individually mark each distress.

#### 2.5.2. Extracting Angle Features

Angle feature extraction begins with fitting a minimum bounding ellipse to the distress region [29]. Crack orientation was determined by fitting the minimum enclosing ellipse via second-order central moments, where the deflection angle was defined as the angle between the ellipse major axis and the image *x*-axis, as shown in Equations (4)–(10).

**Figure 7:**
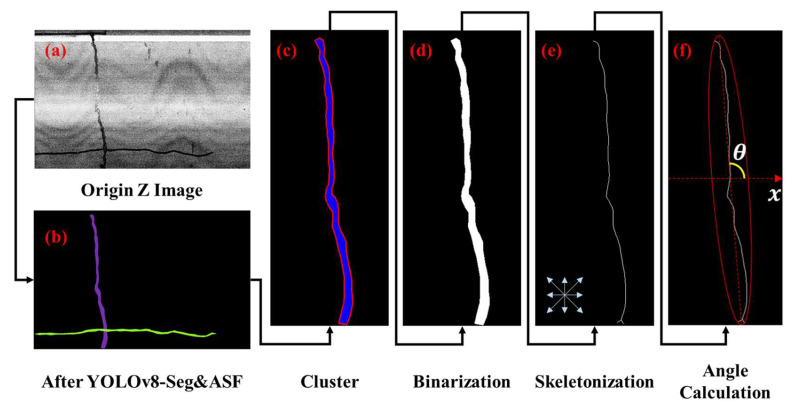
Distress feature extraction process: (**a**) Origin Z Image; (**b**) Object detection image; (**c**) Cluster image; (**d**) Binarization image; (**e**) Skeletonization image; (**f**) Angle calculation.

Deflection angle of the target can be conveniently obtained by approximating the angle between the major axis of the ellipse and the *x*-axis, as shown in Figure 7. θ∈[0,π) can be calculated using Equations (12) and (13). The proposed algorithm was implemented in Python (v3.12.5) using the SciPy and OpenCV scientific computing library.

(4) $$M_{00}=\sum_{x}\sum_{y}I(x,y)$$

(5) $$M_{10}=\sum_{x}\sum_{y}x\cdot I(x,y),M_{01}=\sum_{x}\sum_{y}y\cdot I(x,y)$$

(6) $$\bar{x}=\frac{M_{10}}{M_{00}},\bar{y}=\frac{M_{01}}{M_{00}}$$

(7) $$\mu_{20}=\sum_{x}\sum_{y}{\left(x-\bar{x}\right)}^{2}I(x,y)$$

(8) $$\mu_{02}=\sum_{x}\sum_{y}{\left(y-\bar{y}\right)}^{2}I(x,y)$$

(9) $$\mu_{11}=\sum_{x}\sum_{y}\left(x-\bar{x})(y-\bar{y})I(x,y\right)$$

(10) $$\lambda_{1,2}=\frac{\mu_{20}+\mu_{02}}{2M_{00}}\pm \frac{1}{2}\sqrt{{\left(\frac{\mu_{20}-\mu_{02}}{M_{00}}\right)}^{2}+4{\left(\frac{\mu_{11}}{M_{00}}\right)}^{2}}$$

(11) $$a=\sqrt{\chi^{2}\cdot \lambda_{1}},b=\sqrt{\chi^{2}\cdot \lambda_{2}}$$

(12) $$\theta =\frac{1}{2}\arctan2(2\mu_{11},\mu_{20}-\mu_{02})$$

(13) $$\theta^{\circ }=\theta \cdot \frac{180^{\circ }}{\pi }$$

where I(x,y)=1 indicates pixel belonging to cracks. $\lambda_{1,2}$ correspond to the eigenvalues in the long axis and short axis directions, respectively. Scaling factor is represented by $\chi^{2}$ (standard factor of the smallest circumscribed ellipse in the closed region).

## 3. Results and Discussion

### 3.1. Height Measurement Precision

**Figure 8:**
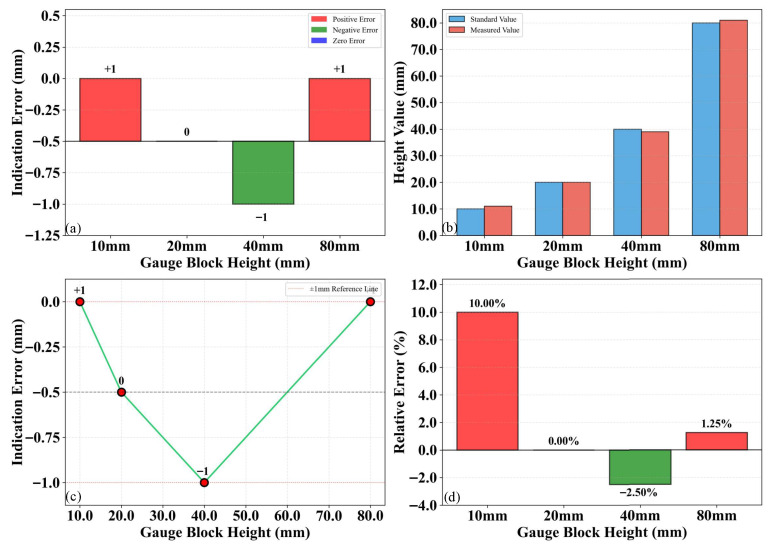
Height measurement: (**a**) Detection error; (**b**) Detected value; (**c**) Error trend line; (**d**) Error percentage.

According to the calibration method described in Section 2.2, the height measurement precision of the 3D laser point cloud was calibrated. Standard blocks with heights ranging from 10 mm to 80 mm were employed for the experiment. To further evaluate 3D laser point cloud system’s performance, a statistical analysis of the measurement errors was conducted in accordance with the Specifications for Automated Pavement Condition Survey (JTG E61-2014). Through repeated measurements, the detection precision was calculated, and the variation in detection error with elevation was analyzed to assess the system’s stability. The performance metrics for height measurement included the absolute error, measured value, error trend line, and error percentage. The detection results are illustrated in Figure 8.

As illustrated in Figure 8, the measurement errors of the 3D laser point cloud ranged from −1 mm to 1 mm. This result reveals that the measurement error of the 3D laser point cloud is less than or equal to 1 mm, which is well below the allowable error specified by the standard, thereby further verifying the accuracy and reliability of the proposed system. Specifically, Figure 8b demonstrates that this error range remained consistent across different elevations. Regarding the relative error shown in Figure 8d, the maximum observed value was 10%. Notably, the measurement error exhibited a negative correlation with target height; for heights between 20 mm and 80 mm, the error remained below 2.5%. These results demonstrate that the 3D laser point cloud measurement system possesses high precision and robust stability.

### 3.2. Planar Measurement Precision

**Figure 9:**
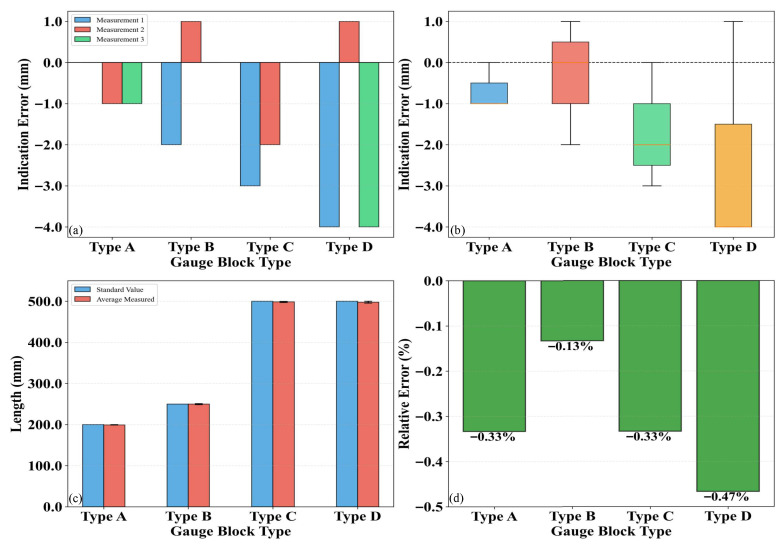
Length measurement: (**a**) Detection error; (**b**) Error distribution; (**c**) Detected value; (**d**) Error percentage.

**Figure 10:**
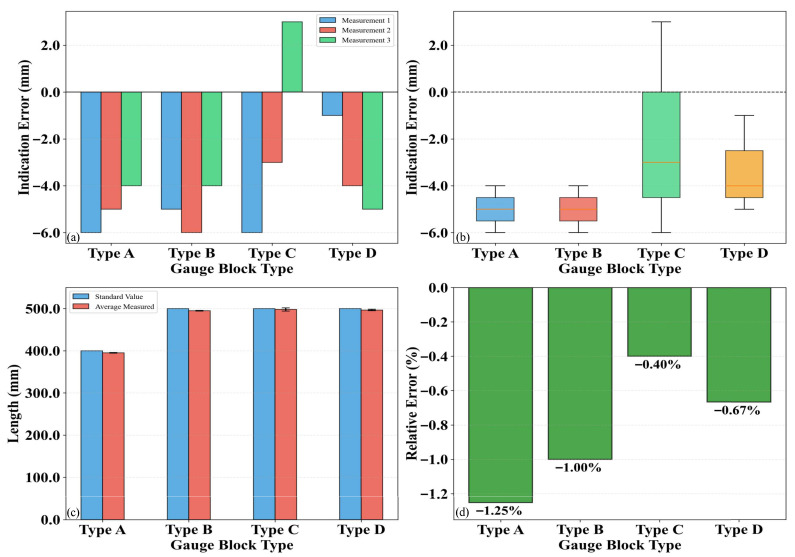
Width measurement: (**a**) Detection error; (**b**) Error distribution; (**c**) Detected value; (**d**) Error percentage.

To calibrate the planar measurement precision of the 3D laser point cloud, the length and width of various standard blocks were measured. The performance metrics for planar measurement included detection error, error distribution, measured value, and relative error. The calibration results are presented in Figure 9 and Figure 10, respectively. Specifically, an analysis of the planar measurement deviations was conducted by referencing the industry verification regulation, the Vehicular Video Detecting System for Pavement Distress (JJG (Transport) 077-2015). This regulation mandates that both transverse and longitudinal measurement deviations be no greater than 5%. As indicated by the results, the proposed system successfully keeps these deviations within the 5% threshold, demonstrating excellent measurement accuracy.

Figure 9 and Figure 10 illustrate the planar measurement precision analysis for four types of standard blocks (Types A, B, C, and D). The results reveal that Type D exhibited the largest measurement error, with an error range of −4 mm to 1 mm and an average relative error of −0.47%, indicating the lowest measurement precision. Conversely, Type B demonstrated the highest precision, characterized by the narrowest error range (−2 mm to 1 mm) and an average relative error of merely −0.13%. The error distribution analysis indicates that Type D possessed the widest dispersion, whereas Type A showed the most concentrated distribution. Furthermore, a comparison between the standard values and measured values confirms that the deviation for Type D was the most significant, while that for Type A was the minimal. Overall, the planar measurement error of the 3D laser point cloud remained below 0.5%, demonstrating high precision and reliability.

### 3.3. Asphalt Pavement Distress Recognition

**Figure 11:**
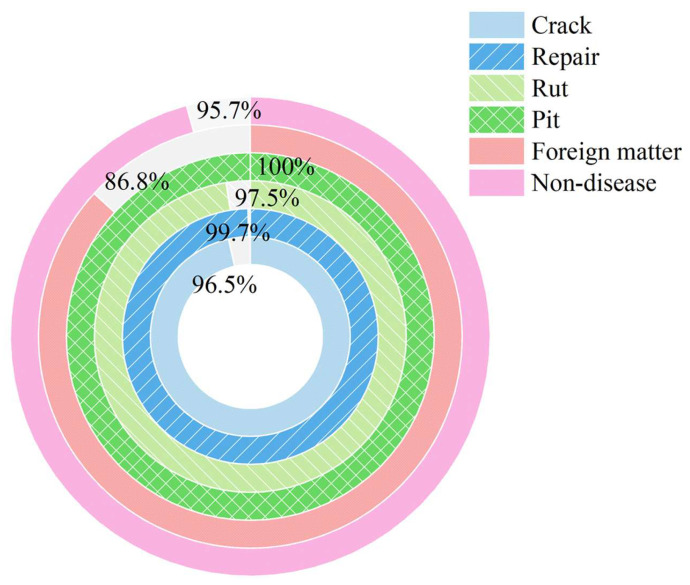
Recall rate of YOLOv8-Seg&ASF model for different distresses.

Figure 11 presents the detection performance of the YOLOv8-Seg&SF model across six pavement distress categories based on 10 independent test runs. The results indicate that the model exhibited stable detection performance for all categories except foreign objects. Specifically, the recall for potholes reached 100%, and the recall rates for other distress types remained above 95%.

Compared to the native YOLOv8-Seg, which achieved an mAP@0.5:0.95 of 0.72, the enhanced YOLOv8-Seg&ASF model demonstrated superior performance with mAP of 0.795. Specifically, the AP@0.5:0.95 for the six categories improved to 0.82, 0.91, 0.82, 0.86, 0.62, and 0.74, respectively.

**Figure 12:**
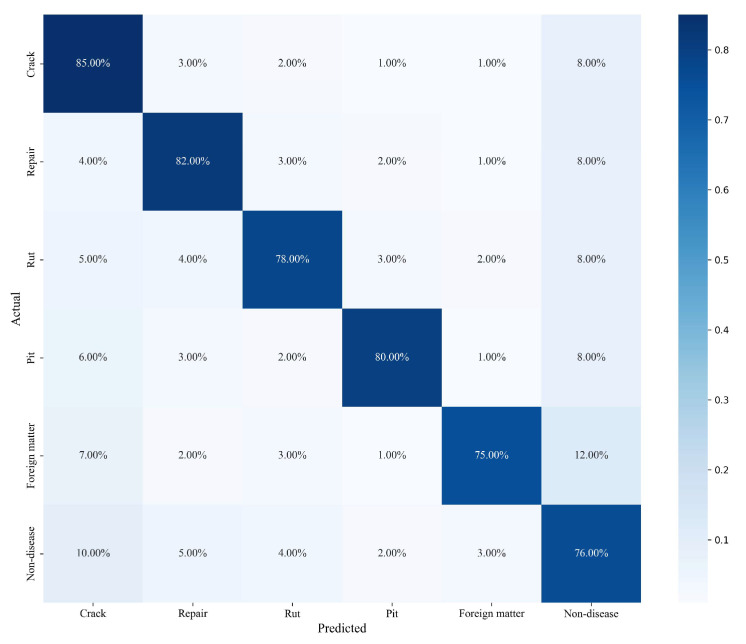
Confusion Matrix for the Validation Set.

The Confusion Matrix for the validation set is presented in Figure 12. The model achieves recognition accuracies of 85%, 82%, 78%, 80%, 75%, and 76% for crack, repair, rut, pit, foreign matter, and non-disease, respectively. Despite cracks, repairs, ruts, and foreign matter accounting for only 5–6% of the data, the model’s recognition rates for these classes remain between 75–85%. Although the sample size is limited, the model successfully captures the distinguishing features of these critical distresses.

**Figure 13:**
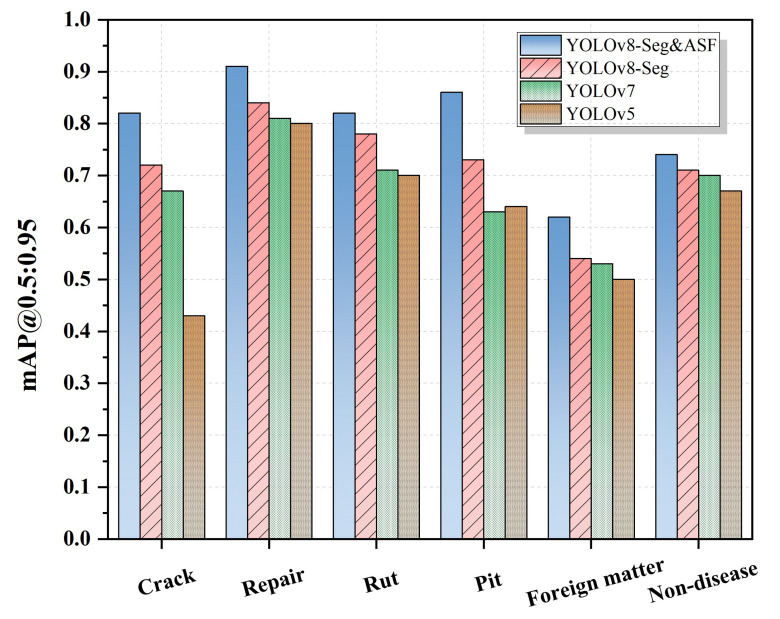
mAP@0.5:0.95 performance between YOLO models.

As shown in Figure 13, the integration of the AFPN structure addressed the limitations of the FPN-PAN network, specifically resolving issues of information loss during the fusion of non-adjacent layers with significant semantic gaps. Furthermore, the asymptotic dynamic fusion mechanism enabled the model to effectively preserve low-level geometric information while incorporating high-level topological context. This resulted in robust multi-scale feature representation, as evidenced by the model’s precision in detecting both fine cracks and extensive rutting.

### 3.4. Asphalt Distress Feature Extraction

This section validates the segmentation data obtained from the YOLOv8-Seg&ASF model and the pavement distress feature extraction method proposed in Section 3.4 through manual verification. The sample preparation procedure described in Section 2.4.1 consisted of two main stages. First, laser scanning equipment was employed to acquire raw point cloud elevation data and determine the precise geospatial coordinates of the samples. Second, based on these coordinates, manual measurements were conducted to determine the actual length and angle of the targets, serving as the ground truth.

**Figure 14:**
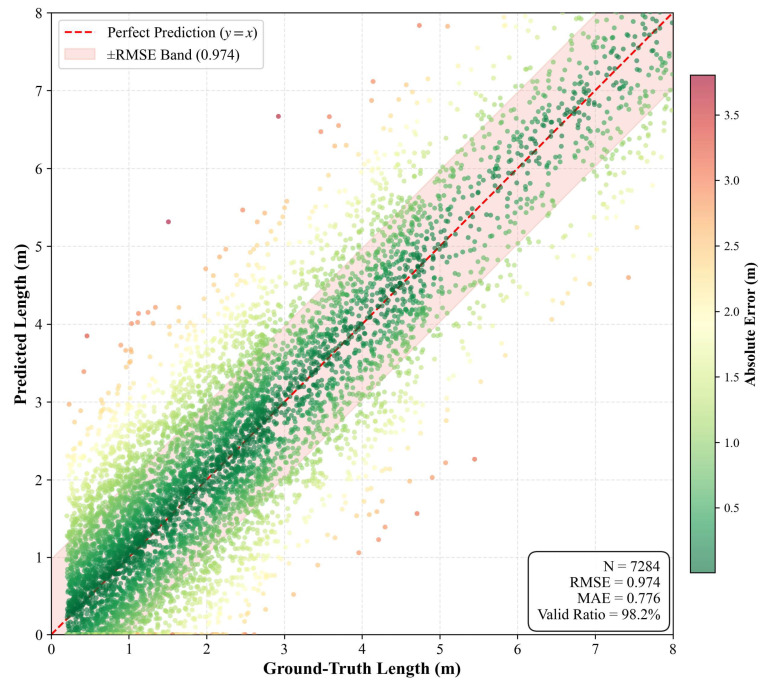
Raw scatter plots.

**Figure 15:**
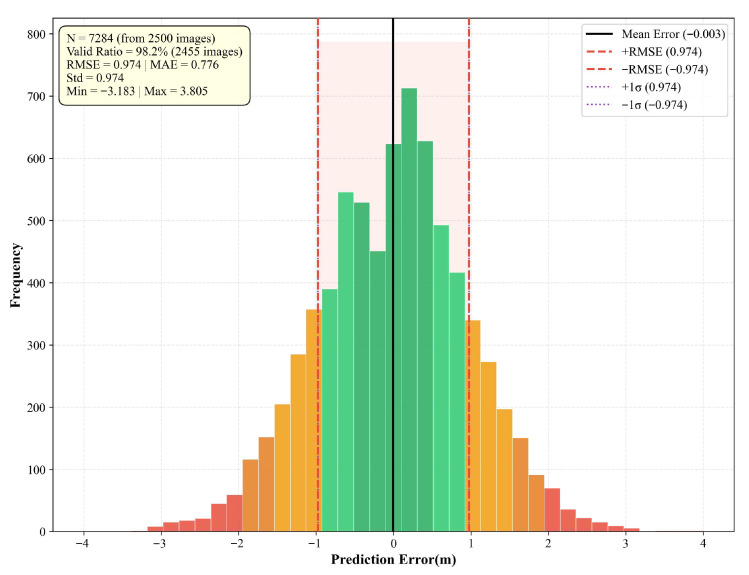
Error distribution histogram.

In the verification phase, the YOLOv8-Seg&ASF model processed the entire dataset to perform segmentation and extraction, yielding prediction targets and corresponding pixel coordinates for all 2500 samples. Acknowledging that the model could not achieve 100% accuracy, the comparative analysis was strictly based on the successfully segmented instances and their corresponding manual measurements. Following three repeated experiments, the average proportion of valid samples was 98.2%, accounting for a total of 7284 detected instances. The original scatter plot (comparing predicted length with ground-truth length) and error distribution histogram of the test set are shown in Figure 14 and Figure 15.

Root Mean Square Error (RMSE) was employed to quantify the deviation between the digital measurement results and manual measurements, as expressed in Equation (14). The RMSE values for the crack angle and length measured by the YOLOv8-Seg&ASF model were 1.687 and 0.974, respectively. These results demonstrate the model’s robust measurement precision for geometric features of pavement distress. The low RMSE values confirm the high reliability of the proposed method in characterizing actual physical scenes.

(14) $$RMSE=\sqrt{\frac{\sum_{i=1}^{n}{\left(X_{obs,i}-X_{pred,i}\right)}^{2}}{N}}$$

Here, RMSE is root mean square error. Specifically, the RMSE for angle is reported in degrees, while the RMSE for length is measured in millimeters. i is the sample number; $X_{obs,i}$ is the manually measured value of the i-th sample; and $X_{pred,i}$ is the digitally calculated value of the i-th sample.

**Figure 16:**
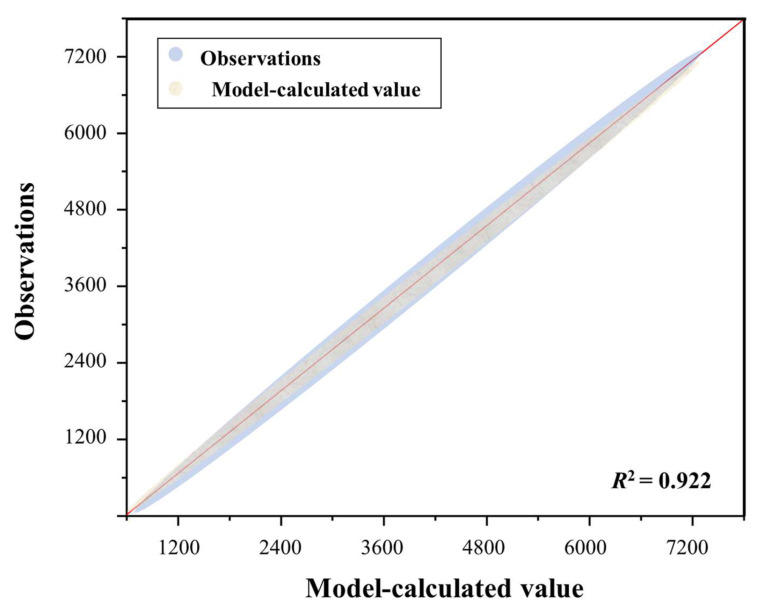
Consistency between observed values and model-calculated values.

Figure 16 demonstrates a coefficient of determination ($R^{2}$) of 0.922 between the observed and predicted values, reflecting high consistency. This finding confirms the reliability of the data acquisition and processing framework. Ultimately, this establishes a robust basis for advancing digital detection methods from identifying distress to quantitatively characterizing distress features.

## 4. Conclusions

This paper proposed a pavement distress recognition method based on 3D laser point cloud data. The three-dimensional laser point cloud system was first calibrated. Subsequently, high-precision 3D laser point cloud scanning was conducted to capture the asphalt pavement profile, and a grayscale compression algorithm was employed to map the point cloud data onto planar images. Finally, by integrating the YOLOv8 object detection algorithm, efficient identification of asphalt pavement distress was achieved. Experimental results demonstrate that the model exhibits robust performance, with an overall recall rate exceeding 85%, a recall rate of 100% for pavement potholes, and a mean Average Precision (mAP@0.5:0.95) of 79.5%. Furthermore, the study successfully extracted geometric features of distress, including length and angle. Comparative analysis against manual measurements yielded Root Mean Square Error (RMSE) values of 1.687 for angle and 0.974 for length, validating the high precision of the extraction method. However, the current dataset comprises only 2500 samples, which is relatively limited for deep learning and may restrict the model’s generalization in highly complex scenarios. Despite this, the proposed framework exhibits strong scalability, allowing for seamless integration with larger datasets and advanced algorithms in the future. Additionally, the grayscale compression significantly reduces computational overhead, and coupled with the efficient inference of YOLOv8, the overall framework demonstrates high computational efficiency suitable for practical engineering applications. These findings provide a novel technical framework for asphalt pavement distress detection, significantly enhancing both detection precision and efficiency.

## Data Availability

The data supporting the findings of this study are available from the corresponding author upon reasonable request due to restrictions from the transport authority’s data privacy policy.
